# China’s Pharmaceutical Ascent: Opportunity for Global Health, Test for US Leadership

**DOI:** 10.7759/cureus.105407

**Published:** 2026-03-17

**Authors:** Arya Babul, Parisa Mahdavi, Momina Hussain, Najib Babul

**Affiliations:** 1 Biomedical Sciences, Society for Awareness of Neglected Diseases, Las Vegas, USA; 2 Biomedical Sciences, West Career and Technical Academy, Las Vegas, USA; 3 Drug Development, Tavolar LLC, Las Vegas, USA; 4 Genomics, Chinese Academy of Tropical Agricultural Sciences, Sanya, CHN; 5 Drug Development, Quadra Therapeutics, Las Vegas, USA

**Keywords:** ai‑enabled drug discovery, china pharmaceutical innovation, fda regulatory policy, global drug development, nmpa, out‑licensing, pharmaceutical regulation, public health policy, regulatory harmonization, us-china relationship

## Abstract

Diseases know no borders; neither should the solutions. - Sir George Alleyne, Address to the Pan American Health Organization, 1998

China’s rapid expansion in pharmaceutical innovation has prompted analyses that variously portray this rise as a geographic shift, a regulatory challenge, or a geopolitical threat. Drawing on recent contributions from Kinch et al., Vokinger et al., Gautam, and Gottlieb, this commentary examines how broader discussions of China’s rise often conflate geography with geopolitics, obscuring the more consequential structural transformation underway in global drug discovery, development, and regulation.

China’s ascent reflects regulatory reform, AI‑enabled discovery, and the out‑licensing of high‑value clinical assets that increasingly shape multinational R&D pipelines. Although geopolitical tensions around data integrity and market access are real, they should not eclipse opportunities for regulatory cooperation, shared standards, and improved patient access. Meanwhile, US vulnerabilities arise less from China’s progress than from domestic policy decisions that weaken scientific capacity and global health partnerships.

A structural, evidence‑based framing, rather than one rooted in rivalry, offers a more constructive foundation for policy, emphasizing regulatory quality and sustained investment in US biomedical infrastructure. The organizing principle for global drug innovation should be health, not geopolitical competition.

## Editorial

Introduction: A debate framed too narrowly

China’s expanding pharmaceutical innovation capacity has prompted a wave of analyses that alternately frame the phenomenon as a shift in scientific geography, a regulatory stress test, or a source of strategic concern. Four recent contributions, Kinch et al.’s empirical mapping of investigational drug origins [1], Vokinger et al.’s examination of regulatory and access implications [2], Gautam’s analyses of China’s growing contribution to global first‑in‑class and best‑in‑class pipelines [3,4], and Gottlieb’s warning about the erosion of US biomedical leadership [5], capture these perspectives with clarity. A complementary perspective comes from Lee and Qian, who examine how US-China scientific interdependence is increasingly reframed through a geopolitical lens [6]. Their analysis underscores the risks of interpreting structural shifts in innovation capacity as strategic threats rather than as features of a globally integrated research ecosystem.

Taken together, however, these analyses reveal a deeper tension: the tendency to interpret China’s scientific and industrial maturation through a geopolitical lens, even when the underlying evidence reflects structural changes in global innovation rather than strategic confrontation. What is often missing is a framework that distinguishes scientific geography from geopolitical intent and recognizes how regulatory reform, AI‑enabled discovery, and the rapid out‑licensing of innovative clinical assets are reshaping global drug development in ways that are fundamentally interdependent.

This commentary aims to clarify that distinction and to examine how mischaracterizing China’s ascent as a geopolitical threat risks obscuring opportunities for regulatory cooperation, scientific exchange, and improved patient access worldwide. The sections that follow examine these dynamics across scientific capacity, regulatory alignment, AI‑enabled discovery, and the evolving geography of global R&D. Our intention is not to diminish the enduring strengths of the US biomedical ecosystem, but to situate China’s rise within a broader, globally interdependent landscape.

When geography is mistaken for geopolitics

Kinch et al.’s Geopolitical Contributions to Pharmaceutical Innovation provides one of the most comprehensive assessments to date of the geographic distribution of investigational new drugs introduced between 2000 and 2024 [1]. Their analysis documents a striking rise in Chinese contributions, driven by both private‑sector growth and an extraordinary surge in public sector activity. By 2023-2024, China had reached parity with the United States in the number of experimental medicines entering clinical testing. Over the past five years, Chinese biotechnology companies have developed more than 600 first‑in‑class drug candidates [7]. A report from the National Medical Products Administration (NMPA), China’s drug regulatory authority, indicates that it approved 48 first‑in‑class drugs across roughly 20 therapeutic areas, some ostensibly superior to products originating from the US, EU, and Japan [8].

These findings are significant, but they should not be mistaken for a geopolitical critique. The focus is on the *where *and *who *of innovation, not on how a state might weaponize pharmaceutical capacity for leverage. Labeling these patterns as “geopolitical contributions” risks conflating distinct domains. Geography becomes geopolitics; industrial policy becomes strategic rivalry. Such linguistic escalation encourages readers to interpret structural shifts in innovation capacity as deliberate acts of statecraft, even when the evidence points to routine scientific maturation.

Precision in terminology is not pedantic; it is essential for avoiding category errors that distort both analysis and policy. This conceptual slippage matters because it encourages zero‑sum interpretations of trends that are, in many respects, positive‑sum. A global increase in first‑in‑class and high‑value therapeutics is a public health gain, not a geopolitical loss.

China’s expanding scientific footprint: a structural transformation

China’s rise in pharmaceutical innovation is rooted in a broader expansion of its scientific capacity. Over the past decade, China has become the world’s largest producer of peer‑reviewed scientific publications, surpassing the United States in total output and showing the fastest growth across STEM fields, according to the US National Science Foundation’s Science and Engineering Indicators [9].

China now leads global scientific output across nearly every major indicator of research quality, not only volume: it surpassed the United States in elite publications in 2024, producing 37,273 articles in the world’s top 145 journals versus America’s 31,930, a “stunning 17% advantage” in what the Nature Index calls the gold standard of scientific excellence (“China achieved what seemed impossible … overtaking the United States in the number of publications appearing in the world’s most prestigious scientific journals”). China also generates the world’s largest share of highly cited papers, with 32% of the top 1% most cited articles, compared with 24% for the United States, reflecting a structural shift toward quality‑driven leadership rather than displacement [10].

Parallel trends are evident in biotechnology patents: China files more international patent applications in pharmaceuticals and biologics than any other country, a pattern highlighted in recent assessments of critical technologies by the Information Technology and Innovation Foundation [11].

**Figure 1:**
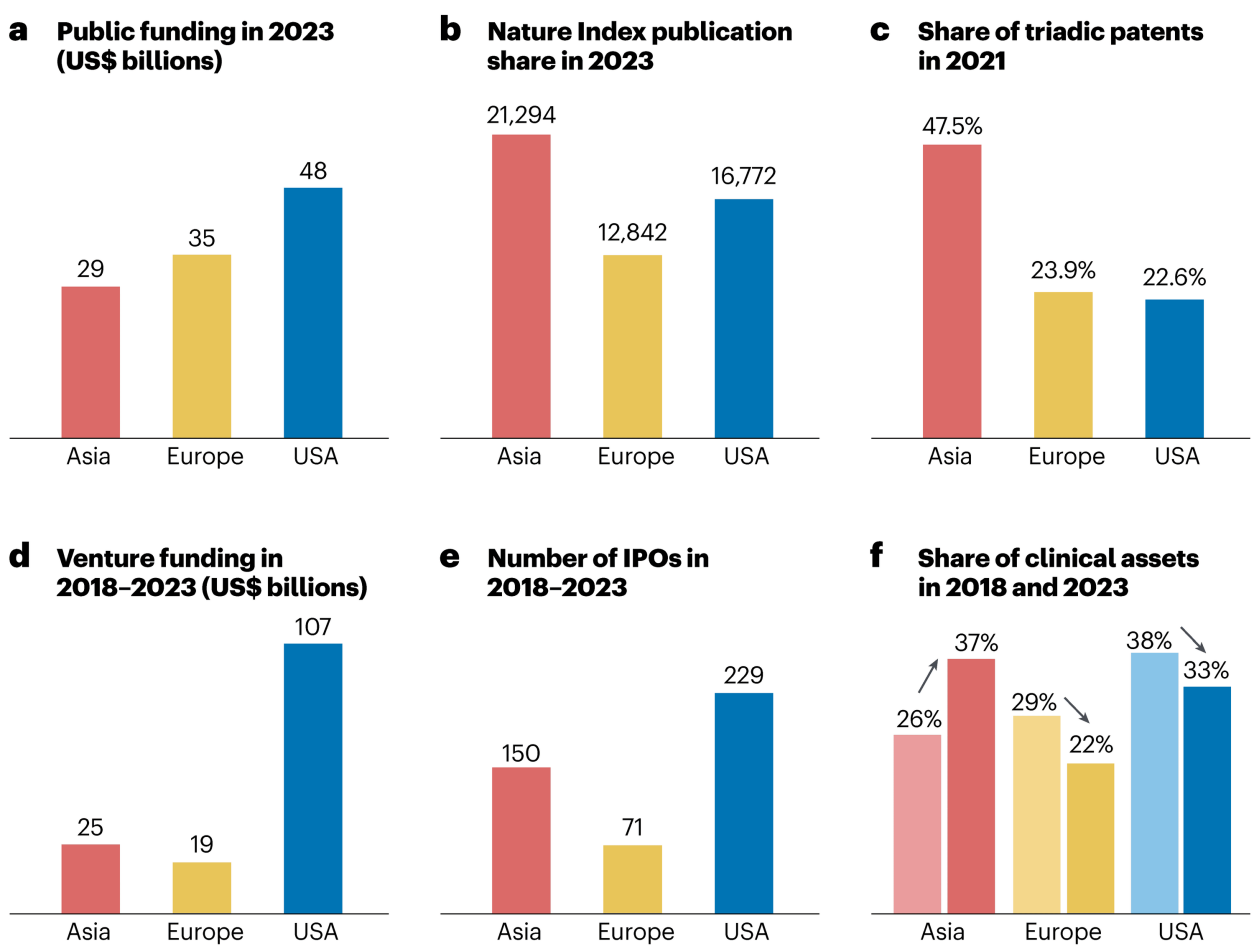
Biomedical Innovation Indicators Across Asia, Europe, and the United States Panels a-f summarize six comparative indicators of biomedical innovation across Asia, Europe, and the United States from 2014 to 2023, including public investment in biomedical R&D, scientific publication output, patent activity, venture financing, initial public offerings, and the number of clinical-stage assets. The figure illustrates Asia’s rapid growth across upstream and downstream innovation metrics, with China emerging as a major contributor to global clinical-stage pipelines. Reproduced with attribution from Gautam [4] in accordance with Springer Nature’s reuse policy

Asia’s scientific output is now approaching US levels across key indicators, including patent activity, peer‑reviewed publications, and the number of clinical‑stage assets (Figure 1).

China, in particular, has built a mature innovation ecosystem anchored by strong academic and translational research institutions, sustained public investment in biomedical R&D, and a growing cohort of domestic biopharma companies advancing first‑in‑class and best‑in‑class candidates.

Nearly 90 China‑origin new molecular entities are currently in phase 3 trials, and seven have secured US FDA approval over the past decade. Although venture funding cycles and capital market conditions present short‑term headwinds, the substantial investments made in recent years position China to remain a significant source of biomedical innovation in the decade ahead [4].

China’s ascent is also deeply embedded in the global pharmaceutical supply chain. As Lee and Qian note, “industry analysts estimate that China now accounts for 70%-95% of the global supply chain for many essential pharmaceutical products” and that the country “is now a global leader when it comes to drug development and manufacturing” [6].

Yet they emphasize that “although China’s advances in biotech and pharma are impressive, the country is still far from being a self‑sufficient biotech superpower,” underscoring that supply chain dominance and manufacturing scale do not obviate continued interdependence in frontier science, regulatory validation, and capital [6].

**Figure 2:**
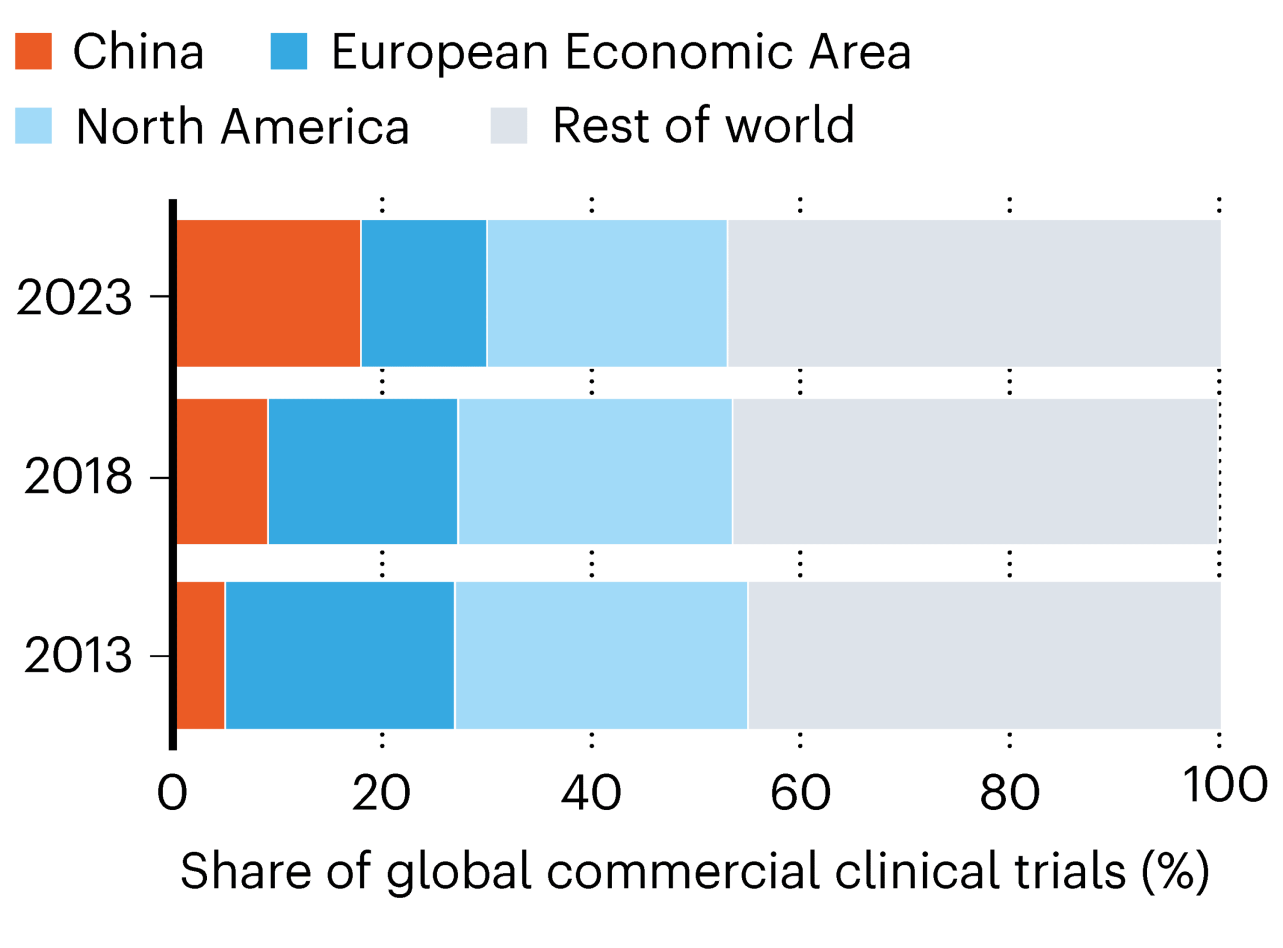
China’s Share of Global Commercial Clinical Trials, 2013-2023 China’s share of industry-sponsored clinical trials increased from 9% in 2018 to approximately 20% in 2023, driven by centralized hospital networks, operational efficiency, and regulatory reform. The figure highlights China’s growing role in global clinical development activity. Reproduced with attribution from Lee and Qian [6] in accordance with Springer Nature’s reuse policy

Recent clinical development metrics reinforce this structural shift. In 2018, China accounted for only 9% of industry‑sponsored clinical trials; by 2023, it was responsible for “about one‑fifth of such trials,” reflecting the pull of large, centralized hospital networks and lower operational costs (Figure 2) [6].

**Figure 3:**
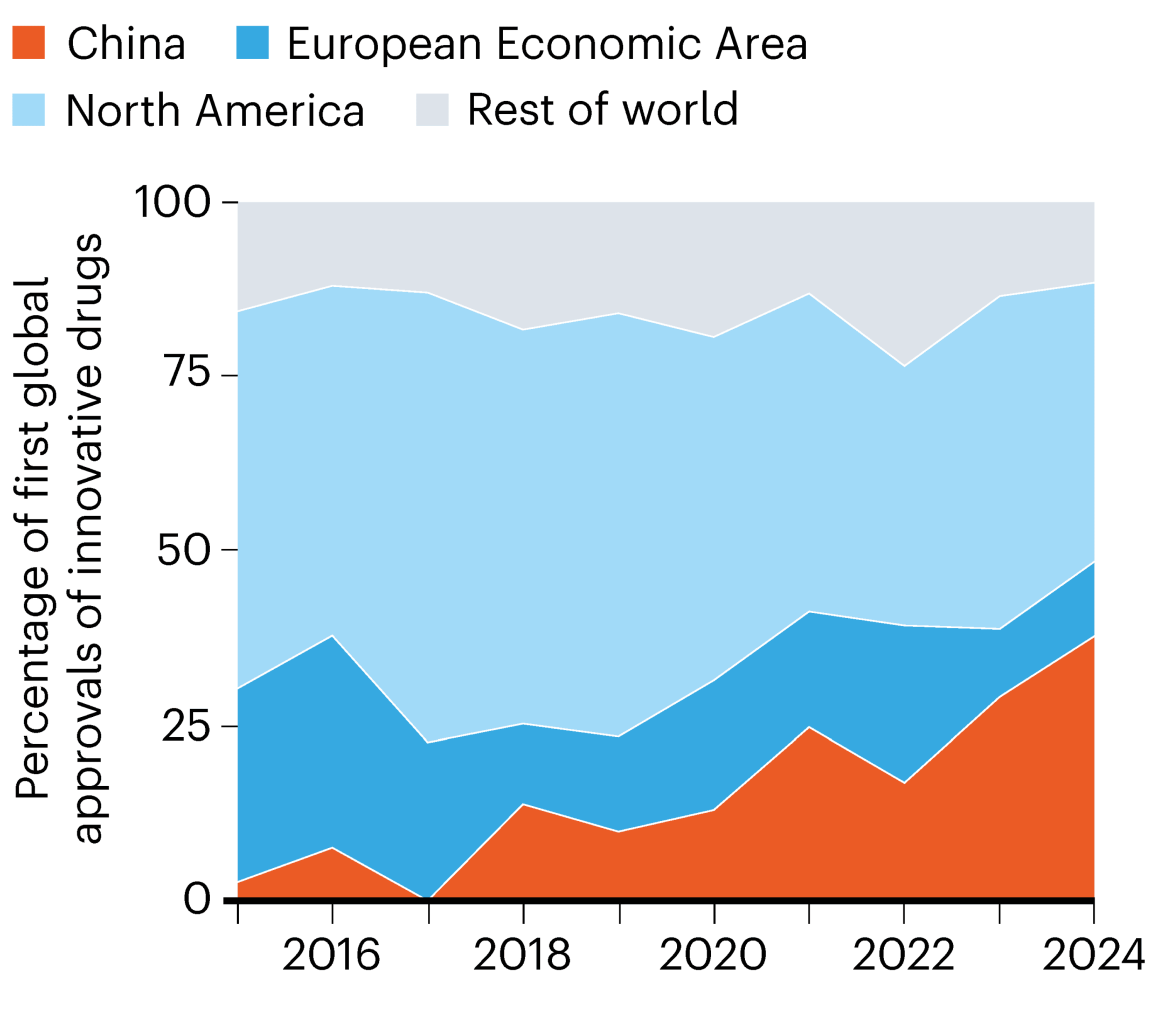
First Global Approvals of Innovative Drugs by Region, 2016-2024 Trends in first-in-world regulatory approvals of innovative drugs across major regions from 2016 to 2024. China’s contribution has increased steadily over the past decade, reflecting the maturation of its innovation ecosystem and alignment with international regulatory standards. Reproduced with attribution from Lee and Qian [6] in accordance with Springer Nature’s reuse policy

Over the past decade, China has also contributed a growing share of first global approvals of innovative drugs, moving from a marginal player to an “important contributor to the number of new drugs approved for use by medical regulators worldwide” (Figure 3) [6]. These trends are consistent with a maturing innovation ecosystem rather than a transient surge.

Taken together, these upstream indicators show that China’s ascent is driven not by geopolitical maneuvering, but by the steady accumulation of scientific capacity, investment, and talent, and that the proliferation of high‑quality publications and patents expands the global reservoir of knowledge that fuels drug discovery, reinforcing the argument that China’s rise represents a structural shift toward interdependence rather than a zero‑sum threat.

Where geopolitics does enter the picture

Vokinger et al.’s The Rise of Drug Innovation in China-Implications for Patient Access in the United States and Globally situates China’s rise within a context where geopolitical tensions are explicit and consequential [2]. Their analysis highlights escalating US-China trade frictions, proposed legislative restrictions on collaboration with Chinese firms, and the risk that political disputes could disrupt cross‑border flows of medicines and data. These are genuine geopolitical dynamics, and they carry real implications for patient access.

The US Biosecure Act, signed into law in late 2025, crystallizes these tensions by barring US pharmaceutical companies that receive federal funding from working with designated Chinese biotech firms [6]. Lee and Qian describe how such measures, combined with longstanding concerns about data handling, have prompted some Chinese officials and industry stakeholders “to advocate for building a closed ‘secure’ biotech ecosystem in China,” including a domestically insulated “closed loop” of contract research organizations, manufacturers, regulators, and payers [6].

This reciprocal securitization illustrates how loosely framed geopolitical narratives can harden into policy proposals that would fragment, rather than govern, an already interdependent system.

Yet even in this setting, the authors emphasize the potential for constructive engagement. China’s alignment with international regulatory standards, its participation in global harmonization initiatives, and its growing portfolio of first‑approved and first‑in‑class drugs create opportunities for regulatory cooperation. Initiatives such as Project Orbis could, if expanded to include China, accelerate access to promising therapies for nearly half of the world’s cancer patients.

The message is clear: geopolitical tensions are real, but they need not dictate the trajectory of global drug innovation. Regulatory collaboration, mutual trust, and evidence‑based evaluation of foreign data offer a path toward shared benefit.

China’s progress in drug discovery reflects the same structural maturation seen across its other high‑technology sectors. Yet, as Vokinger et al. emphasize, Chinese drugmakers still confront market access barriers that resemble the transitional challenges previously faced by other emerging innovators.

Beyond their oncology examples, ivarmacitinib, a selective oral Janus kinase (JAK) 1 inhibitor developed by Reistone Biopharma and Jiangsu Hengrui Pharmaceuticals, received Chinese regulatory clearance in 2025 for ankylosing spondylitis, rheumatoid arthritis, atopic dermatitis, and alopecia areata [12].

Severe alopecia areata is a psychologically and socially devastating autoimmune disease, and effective systemic therapies remain limited. Ivarmacitinib, a China‑originated JAK1 inhibitor, was recently compared with deuruxolitinib, a US‑originated deuterated JAK1/JAK2‑selective inhibitor previously identified as having the highest efficacy among US‑approved JAK inhibitors in network meta‑analyses for severe alopecia areata [13,14]. These two agents are commercialized exclusively within distinct pharmaceutical innovation ecosystems. In Bayesian network meta‑analyses, ivarmacitinib yielded higher odds of achieving clinically meaningful scalp hair regrowth [14]. These findings support its potential best‑in‑class positioning and underscore the global benefits of therapeutics discovered or developed in different geographies.

The costs of the bogeyman narrative

Former FDA Commissioner Gottlieb’s perspective, Securing America’s Pharmaceutical Innovation Edge, is the most overtly strategic, warning that US underinvestment in the NIH and strain on the FDA threaten to erode America’s biomedical leadership at a moment when China is rapidly advancing [5]. Now a senior fellow at the American Enterprise Institute, Gottlieb approaches the issue from a vantage point shaped by national security‑oriented policy analysis.

His concerns about domestic institutional fragility are well-founded. Erosion of NIH funding capacity and reductions in FDA staffing undermine the infrastructure that has historically enabled the US to set global standards for scientific rigor, ethical norms, and regulatory excellence.

We agree with Gottlieb’s observation that “showing that a drug is safe and effective through rigorous clinical studies, conducted under the FDA’s oversight, gives the US a competitive edge that China cannot match,” and that “the data from their later‑stage, human trials are still viewed skeptically by US regulators and drug companies.”

Chinese pharmaceutical companies continue to face challenges in securing timely market access in the US, largely due to FDA concerns regarding data integrity and regulatory compliance.

In addition, US regulation 21 CFR 314.106(b) requires that foreign data be applicable to the US population and medical practice, and that such data be considered valid without the need for an onsite inspection, or, if an inspection is required, that the FDA be able to validate the data through appropriate means [15].

However, China’s regulator, the National Medical Products Administration (NMPA), has joined the International Council for Harmonization (ICH) and the Pharmaceutical Inspection Cooperation Scheme (PIC/S), whose mandates include harmonizing global standards for drug development and manufacturing. These barriers, which echo earlier temporary hurdles faced by other countries seeking US approval, will not be sufficient to preserve US dominance in the long term.

The deeper issue is the framing of China as a looming adversary. This Cold War-era mobilization narrative is strategically self‑defeating and obscures the core problem: the greatest threat to US biomedical leadership is not China’s rise but the erosion of domestic scientific and regulatory capacity. No amount of geopolitical rhetoric can substitute for sustained investment in research, regulatory modernization, and the academic ecosystems that generate early‑stage discovery.

Moreover, casting China as a bogeyman can lead to blunt, counterproductive policies: indiscriminate restrictions on collaboration, reflexive skepticism toward foreign clinical data, or trade measures that disrupt supply chains and raise costs for patients. These approaches may satisfy political instincts but do little to strengthen scientific capacity or improve public health.

China’s rise is a wake‑up call, not a red flag. We do not need China as a rhetorical foil to identify the real challenges confronting US pharmaceutical innovation or to pursue the thoughtful solutions Gottlieb outlines.

AI-enabled discovery: A missed opportunity in geopolitical narratives

A rapidly expanding dimension of China’s pharmaceutical ascent, and one that receives too little attention in geopolitical narratives, is the country’s emergence as a global hub for AI‑enabled drug discovery. Chinese biotech firms such as XtalPi, Insilico Medicine, Helixon, and CSPC have become preferred partners for Western pharmaceutical companies seeking faster, more computationally sophisticated approaches to early‑stage R&D [16]. Recent multibillion‑dollar licensing and co‑development agreements with AstraZeneca, Pfizer, Sanofi, and Eli Lilly illustrate that China is no longer merely a geographic competitor; it is an increasingly indispensable collaborator in next‑generation drug discovery technologies.

These partnerships are not acts of geopolitical defection; they are rational responses to scientific opportunity. They reflect the reality that AI‑driven discovery pipelines, large patient datasets, and advanced automation capacity are now distributed globally, and that no single country holds a monopoly on the tools that will define the future of therapeutic innovation.

China’s AI‑enabled drug discovery sector benefits from structural advantages: a deep and relatively inexpensive talent pool spanning chemistry, engineering, and AI; a state‑backed startup ecosystem; targeted government investment, including the designation of AI drug discovery as a priority in the 2025 Five‑Year Plan; and massive clinical and genomic datasets enabled by a national insurance system covering more than 600 million people [16].

Many firms have integrated advanced automation and robotics into their R&D workflows, accelerating candidate screening and reducing development costs.

These features, combined with streamlined regulatory pathways and growing private sector investment from technology giants such as Tencent and ByteDance, give Chinese AI‑biotech companies a cost‑efficient, data‑rich, and highly scalable innovation environment.

Rather than reinforcing a narrative of rivalry, the rise of China’s AI‑biotech ecosystem underscores the potential for mutually reinforcing innovation. US and European firms benefit from China’s computational scale, engineering talent, and rapid iteration cycles, while Chinese firms gain access to global regulatory pathways, clinical trial networks, and commercialization expertise.

Attempts to frame these developments as geopolitical threats risk undermining the very partnerships that are driving scientific progress. A more constructive approach would recognize that AI‑enabled drug discovery is inherently transnational and that shared standards, interoperable data systems, and joint regulatory pilots could amplify benefits for patients worldwide.

These advances in AI‑enabled discovery feed directly into the next structural shift: the rapid expansion of out‑licensing that now anchors China’s integration into global R&D pipelines.

Out-licensing and the new geography of global R&D

**Figure 4:**
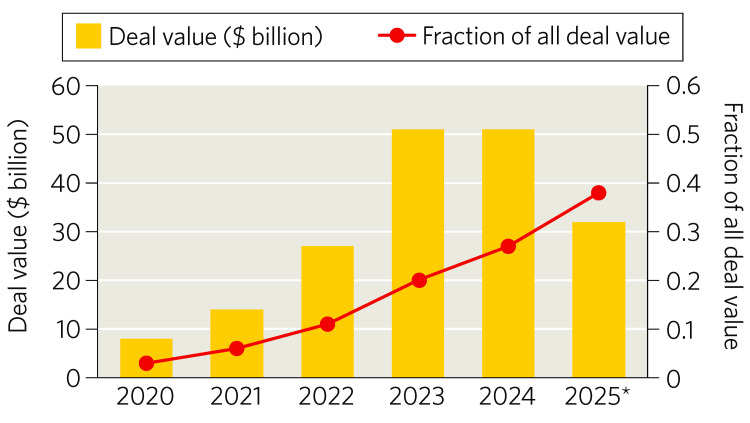
China Licensing Deals, 2020-2025: Chinese Biopharma Assets Account for a Growing Share of Licensing Deals Total annual deal value for licensing agreements involving Chinese-origin assets from 2020 to 2025. Licensing activity expanded more than 10-fold during this period, with 2025 values extrapolated from Q1-Q2 data. The figure illustrates China’s emergence as a major source of high-value, clinic-ready assets for multinational pharmaceutical companies. Adapted from Gautam [3] in accordance with Springer Nature’s reuse policy

Global pharmaceutical licensing and R&D collaboration activity amounted to roughly $170-$180 billion annually between 2020 and 2025. Although total deal value remained relatively steady across these years, one striking shift was the surge in licensing transactions involving China‑originated assets. These deals expanded more than 10-fold (Figure 4), from about $5 billion in 2020 (around 3% of the global total) to more than $50 billion by 2024 (approximately 30%). This sharp rise reflects China’s accelerating innovative capacity. China now represents close to one‑third of all clinical-stage assets worldwide, placing it alongside the United States and well ahead of Europe [3,4].

A complementary dimension of China’s rise in pharmaceutical innovation is the rapid expansion of out‑licensing from Chinese biopharma to US and European pharmaceutical companies. Over the past five years, Western firms have increasingly turned to China not only for AI‑enabled discovery platforms but also for clinic‑ready, innovative therapeutic assets that can be advanced through global development pipelines. Between 2020 and 2025, major multinational companies collectively committed tens of billions of dollars to in‑license Chinese‑origin assets across oncology, immunology, cardiometabolic disease, and cell therapy [3,4].

**Figure 5:**
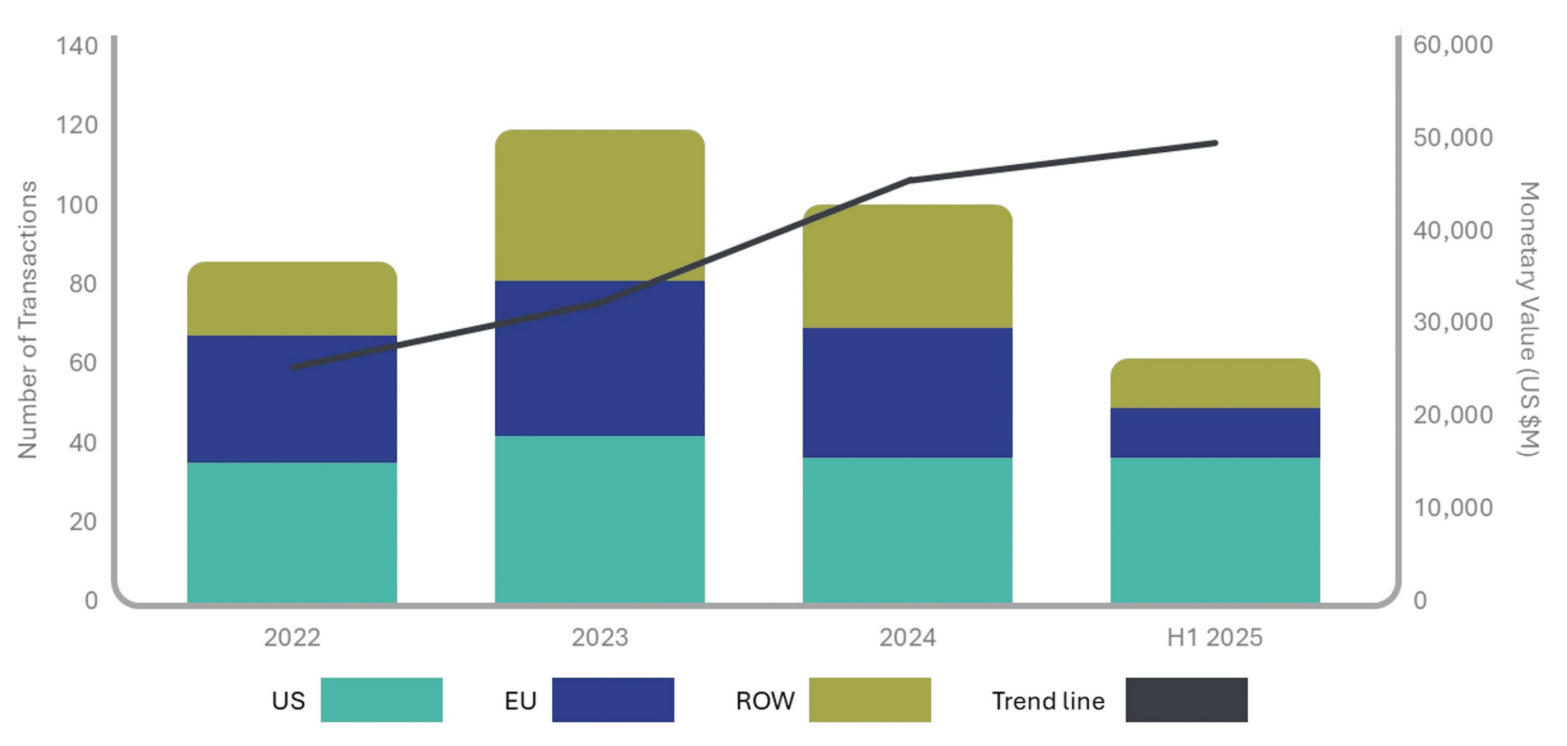
Outbound Partnering Trends for China Origin Assets, 2022-H1 2025 The figure displays annual partnering volume and total potential deal value, highlighting the rapid expansion of China-to-US and China-to-EU licensing activity. Adapted from Haggerty and Ahmed [17]

Deal‑level data from IQVIA’s Pharma Deals database underscore the scale and trajectory of this shift. Haggerty and Ahmed report that “61 China‑to‑International partnering deals were announced in H1 2025, with 37 of those involving a US company,” and that aggregate potential deal value for Chinese‑origin assets reached US$48.5 billion in the first half of 2025 alone, surpassing the US$44.8 billion recorded for all of 2024 (Figure 5) [17]. Notably, 61% of outward partnering deals in H1 2025 were with US‑headquartered companies, compared with 37% in 2024, reflecting both growing confidence in Chinese assets and intensifying reliance on them to replenish global pipelines [17].

The structure of these alliances further supports a structural, opportunity-driven interpretation rather than a geopolitical one. According to IQVIA, companies “have started to show preference for forming heavily back-loaded cross-border partnerships with China headquartered firms, often relying on them to carry out efficient early stage trials in China, and only investing further in global trials once the asset has been sufficiently de-risked” [17]. This pattern, front-loading scientific and operational risk in China while preserving global commercialization options, illustrates how interdependence is being operationalized through contract design, not merely rhetoric. This pattern of reciprocal benefit directly contradicts narratives that portray China’s rise as a zero-sum threat. Instead, it demonstrates that global drug innovation increasingly depends on cross-border exchange, with China emerging as a key supplier of differentiated clinical candidates that complement US and European strengths in regulatory science, commercialization, and late-stage development.

This surge reflects a structural shift: Chinese firms are no longer peripheral participants in global drug development. They have become primary contributors of high‑value, de‑risked clinical assets, prompting companies such as Merck, AstraZeneca, Bristol Myers Squibb, Novartis, and Pfizer to execute multibillion‑dollar licensing agreements with Chinese partners [3,4]. These deals reflect rational responses to scientific opportunity, competitive pressure, and looming patent cliffs.

The therapeutic scope of these alliances is broad. While oncology continues to dominate, IQVIA reports rising activity in immunology, inflammatory disease, cardiometabolic programs leveraging RNA interference platforms, and next-generation incretin biology. Recent examples include multibillion-dollar agreements for GLP-1/GIP agonists, PD-1/VEGF bispecifics, and CLDN18.2-targeted ADCs [17]. Out-licensing has also become a critical financing mechanism for Chinese biopharma during periods of constrained domestic capital markets, creating a mutually reinforcing ecosystem in which Western firms gain differentiated assets and Chinese companies gain capital, regulatory experience, and global trial infrastructure. The trajectories of zanubrutinib and toripalimab, both China-origin therapies now FDA-approved, illustrate how cross-border development converts local innovation into global public goods [6].

Policies that restrict collaboration or treat Chinese-origin assets as inherently suspect risk undermining the very partnerships that are accelerating therapeutic progress worldwide. In trying to weaken a rival, we would be weakening our own pipelines.

Healthy rivalry versus destructive competition

A degree of competition between innovation systems can be healthy. It drives efficiency, accelerates discovery, and expands the therapeutic frontier. But competition becomes destructive when it is framed as existential rivalry rather than scientific pluralism.

The United States benefits when China produces high‑value therapeutics that can be rigorously evaluated, licensed, or improved upon. China benefits when US regulatory science sets global standards for safety and efficacy. These dynamics are not mutually exclusive; they are mutually reinforcing.

Even within China, there is recognition that a go‑it‑alone strategy would be self‑limiting. Lee and Qian highlight that a “hypercompetitive corporate culture” has driven multiple firms toward the same molecular targets, with “nearly 40% of registered clinical trials for cell therapies … focused on known molecular targets” between 2021 and 2023, reflecting incremental optimization more than breakthrough discovery [6].

They also note persistent trust deficits, “US intelligence officials reported that Chinese biotech firms had transferred intellectual property of US clients to Chinese authorities without the clients’ consent,” and structural financial constraints, including immature capital markets and a National Reimbursement Drug List that extracted an average 63% price discount in 2024, making it harder for domestic firms to recoup high‑risk R&D investments [6].

These features argue for deeper, not shallower, integration with global science, capital, and regulatory systems.

The danger lies not in competition itself but in allowing geopolitical narratives to convert scientific rivalry into strategic hostility. Such framing undermines the collaborative mechanisms that make global drug innovation possible and risks fragmenting a system that depends on cross‑border exchange.

Self-inflicted weakness in US biomedical leadership

If geopolitical narratives obscure the real drivers of US vulnerability, recent domestic policy decisions illuminate them starkly. For decades, the US exercised global leadership in tropical medicine and infectious disease research through USAID, the CDC, and the NIH, not only via field stations, collaborative trials, and training programs but also through sustained financial support to the World Health Organization and its neglected tropical disease (NTD) initiatives [18]. This ecosystem of partnerships, infrastructure, and institutional memory was a strategic asset as consequential as any technological advantage.

This global health ecosystem was also a profound source of soft power. Through efforts like PEPFAR and US‑led malaria programs, American scientists and clinicians built relationships that shaped health outcomes and cemented US credibility in ways no geopolitical framing could match. By training local scientists, supporting disease control programs, and sustaining long‑term research partnerships, the United States cultivated reservoirs of trust and scientific legitimacy that no messaging campaign could manufacture. In many parts of Africa, Asia, and Latin America, US public health engagement was the most visible and most admired expression of American leadership. Its erosion, therefore, represents not only a loss of technical capacity but also a forfeiture of diplomatic influence.

Yet in recent years, shifts in funding priorities and workforce stability have weakened this foundation. The dismissal of thousands of public health experts across federal agencies and implementing partners has hollowed out the capacity that once enabled the US to shape global health agendas. Philanthropy and private entities have attempted to fill the gaps, but they cannot replace the continuity, scale, and legitimacy of public investment.

The consequences have been immediate and far‑reaching. Johns Hopkins University, long a global center of excellence in tropical medicine, laid off more than 2,200 employees worldwide after the abrupt termination of $800 million in USAID funding [19]. Similar cuts triggered mass furloughs at RTI International and FHI 360, disrupting programs in maternal health, infectious disease, and clean water access [20]. Dozens of field sites have closed, and hundreds of clinical trials across Africa, Asia, and Latin America have been halted or abandoned.

These disruptions are not peripheral to US biomedical leadership; they strike at its core. A recent analysis by Patel et al. in JAMA Internal Medicine found that roughly one in 30 NIH‑funded clinical trials experienced significant disruption, affecting more than 74,000 participants [21]. Trials conducted outside the US, often in areas of infectious disease, prevention, and behavioral health, were disproportionately affected.

At the same time, China has expanded its own global health footprint in precisely these domains, deepening partnerships in infectious disease research, vaccine development, and public health infrastructure across Africa, Asia, and Latin America.

In this context, the narrative of geopolitical rivalry becomes not only misleading but dangerously self‑exculpatory. The US is not being overtaken because China is rising; it is being overtaken because it is dismantling the institutions that once made it indispensable.

The failure of policymakers to wield a scalpel rather than an axe in trimming NIH budgets is a historic misstep that risks dismantling strategic research infrastructure and derailing the careers of emerging scientific talent. Protecting NIH funding is not merely a fiscal decision; it is a strategic imperative for sustaining America’s competitive edge in biomedical innovation for decades to come [22].

Interdependence as the defining reality of modern drug innovation

Across discovery, development, regulation, and commercialization, the through line of China’s pharmaceutical ascent is not geopolitical ambition but structural interdependence. Scientific capacity, clinical trial infrastructure, AI‑enabled discovery platforms, and late‑stage regulatory pathways are now distributed across multiple regions. No single country, neither the United States nor China, controls the full innovation continuum.

This interdependence is not a vulnerability; it is the architecture of modern biomedical progress.

China’s strengths in computational chemistry, automation, and early‑stage clinical execution complement US strengths in regulatory science, global trial networks, and commercialization. European agencies contribute deep expertise in benefit-risk assessment and post‑marketing surveillance. Multinational companies increasingly rely on cross‑border partnerships to de-risk assets, accelerate timelines, and diversify pipelines.

The global drug innovation system is therefore best understood not as a contest of national champions but as a distributed network of capabilities. Attempts to force this system into a Cold War frame, whether through securitization narratives, restrictive legislation, or reflexive suspicion of foreign data, risk undermining the very mechanisms that have expanded therapeutic options for patients worldwide.

The question is not whether China’s rise will reshape global drug development. It already has. The question is whether the United States will respond by strengthening its own scientific institutions and embracing constructive interdependence, or by retreating into a defensive posture that weakens its long‑term competitiveness.

Reframing the narrative

China’s ascent in pharmaceutical innovation is best understood not as a geopolitical provocation but as a structural transformation in the global research ecosystem. The evidence across scientific output, clinical development activity, AI‑enabled discovery, and out‑licensing patterns points to a system in which innovation capacity is increasingly distributed, interdependent, and shaped by cross‑border exchange. Interpreting these shifts through a rivalry‑based lens obscures the deeper reality: global drug development has become a networked enterprise in which no single country can sustain leadership through isolation or defensive policy.

A more accurate framing recognizes that China’s rise has expanded, not displaced, the global reservoir of scientific knowledge and clinical‑stage assets. The proliferation of first‑in‑class candidates, the maturation of regulatory standards, and the rapid growth of AI‑driven discovery platforms have created new opportunities for collaboration that benefit patients worldwide. These developments reflect the natural evolution of a maturing innovation ecosystem rather than a strategic challenge to US interests.

At the same time, the United States retains enduring strengths in regulatory science, translational research, and commercialization. These advantages are reinforced, not threatened, when foreign data are rigorously evaluated, when promising assets are licensed into global pipelines, and when scientific exchange is governed by transparent, evidence‑based standards. The most productive path forward lies not in securitizing scientific progress but in leveraging complementary strengths across systems.

Reframing China’s ascent in structural rather than adversarial terms also clarifies the true sources of US vulnerability. The erosion of domestic scientific capacity, the contraction of global health partnerships, and the underfunding of regulatory agencies pose far greater risks to US biomedical leadership than China’s progress. A narrative that casts China as a monolithic competitor distracts from the policy choices that have weakened the very institutions that once anchored US global influence.

A constructive reframing, therefore, requires moving beyond zero‑sum assumptions and recognizing that global drug innovation is shaped by shared incentives: faster discovery, more efficient development, and broader patient access. Policies grounded in interdependence, rather than rivalry, offer the most credible path to strengthening US leadership while advancing global health.

Conclusion

Both the United States and China contribute essential capabilities to the global innovation system, and recognizing this complementarity is central to the argument we advance. China’s growing role in pharmaceutical innovation reflects structural changes in global science, not a geopolitical threat. Efforts to frame this evolution as adversarial risk undermining the collaborative mechanisms that accelerate therapeutic progress and improve patient access. Sustained US leadership will depend less on countering China and more on reinvesting in scientific capacity, regulatory excellence, and global health partnerships. A narrative grounded in evidence, interdependence, and shared public health goals offers a more constructive foundation for policy than one rooted in rivalry. The future of drug innovation will belong to those who understand that while borders divide nations, they do not divide science.

## References

[1] Kinch MS, Alawi A, Schwartz T, Kraft Z (2026). Geopolitical contributions to pharmaceutical innovation. Drug Discov Today.

[2] Vokinger KN, Li G, Wouters OJ (2025). The rise of drug innovation in China - implications for patient access in the United States and globally. N Engl J Med.

[3] Gautam A (2025). China’s increasing flow of innovative assets into big pharma R&D pipelines. Nature.

[4] Gautam A (2025). Life science ecosystems in Asia: biomedical innovation trends over the past decade. Nat Rev Drug Discov.

[5] Gottlieb S (2025). Securing America’s pharmaceutical innovation edge. JAMA Health Forum.

[6] Lee LC, Qian J (2026). China's biotech boom: why the nation must collaborate to stay ahead. Nature.

[7] Masson G (2026). China biotechs ‘reshaping’ US biopharma as outlicensing deals rise 11%: Jefferies report. https://www.fiercebiotech.com/biotech/china-biotechs-reshaping-us-biopharma-outlicensing-deals-rise-11-jefferies-report.

[8] (2026). Measures facilitate approval of 48 first-in-class innovative drugs. https://tinyurl.com/yjvsatzm.

[9] (2024). Invention, knowledge transfer, and innovation. Science and Engineering Indicators.

[10] Wagner CS (2025). China’s historic rise to the top of the scientific ladder. Quincy Institute for Responsible Statecraft, , Legislative & Policy Note No. 16.

[11] Atkinson RD, Foote C (2025). How China is outperforming the United States in critical technologies. Information Technology & Innovation Foundation.

[12] Keam SJ (2025). Ivarmacitinib sulfate: first approval. Drugs.

[13] Babul A, Mehta D, Soliman Y, Hussain M, Babul N (2025). Comparative efficacy of Janus kinase inhibitors indicated for severe alopecia areata: a Bayesian network meta-analysis and matching-adjusted indirect comparison. J Dermatol.

[14] Babul A, Mehta D, Soliman Y, Hussain M, Babul N (2025). Efficacy of Deuruxolitinib and Ivarmacitinib, Two Recently Available Janus Kinase Inhibitors (JAKIs) for Severe Alopecia Areata (AA): A Bayesian Network Meta-Analysis (NMA) and Matching-Adjusted Indirect Comparison (MAIC). American Academy of Dermatology Annual Meeting, Poster No. 71564, March 27-31, 2026, Denver.

[15] (2026). Code of Federal Regulations: Title 21: §314.106 Foreign data. https://www.ecfr.gov/current/title-21/chapter-I/subchapter-D/part-314/subpart-D/section-314.106.

[16] Liang L (2026). China’s AI drug discovery companies land huge deals with Big Pharma. 6 August.

[17] Haggerty L, Ahmed T (2026). China’s outbound Pharma partnering deals gain momentum. https://www.iqvia.com/library/articles/chinas-outbound-pharma-partnering-deals-gain-momentum.

[18] Babul A, Mahdavi P, Hussain A, Janjua ZH, Hussain M (2025). Reclaiming rural medicine: mass drug administration, community sovereignty, and institutional memory. Cureus.

[19] (2026). In wake of federal funding cuts, Johns Hopkins scales back USAID-supported work around the globe. https://hub.jhu.edu/2025/03/14/johns-hopkins-usaid-funding-cuts-global-health/.

[20] (2026). Durham based FHI 360 announce staff cuts due to lack of funding. https://abc11.com/post/fhi-360-layoffs-federal-funding-nc-nonprofit-announces-staff-reduction-durhamdue-lack/16152917/.

[21] Patel VR, Liu M, Jena AB (2026). Clinical trials affected by research grant terminations at the National Institutes of Health. JAMA Intern Med.

[22] Jalali MS, Hasgul Z (2025). Potential trade-offs of proposed cuts to the US National Institutes of Health. JAMA Health Forum.

